# Supplementary material for: Diverse species-specific phenotypic consequences of loss of function sorting nexin 14 mutations
Source: Sci Rep. 2020 Aug 13;10:13763. doi: 10.1038/s41598-020-70797-2 (PMC7427099; doi:10.1038/s41598-020-70797-2)
Supplement: Supplementary file 1 — Supplementary Information. [file 41598_2020_70797_MOESM1_ESM.docx]

**Diverse Species-Specific Phenotypic Consequences of Loss of Function *Sorting Nexin 14* Mutations**

Dale Bryant, Marian Seda, Emma Peskett, Constance Maurer, Gideon Pomeranz, Marcus Ghosh, Thomas A. Hawkins, James Cleak, Sanchari Datta, Hanaa Hariri, Kaitlyn M. Eckert, Daniyal J. Jafree, Claire Walsh, Charalambos Demetriou, Miho Ishida, Cristina Alemán-Charlet, Letizia Vestito, Rimante Seselgyte, Jeffrey G. McDonald, Maria Bitner-Glindzicz, Myriam Hemberger, Jason Rihel, Lydia Teboul, W. Mike Henne, Dagan Jenkins, Gudrun E. Moore, Philip Stanier

**Supporting Information:**

**Supplemental Figures 1-9**

**Supplemental Tables 1-3**


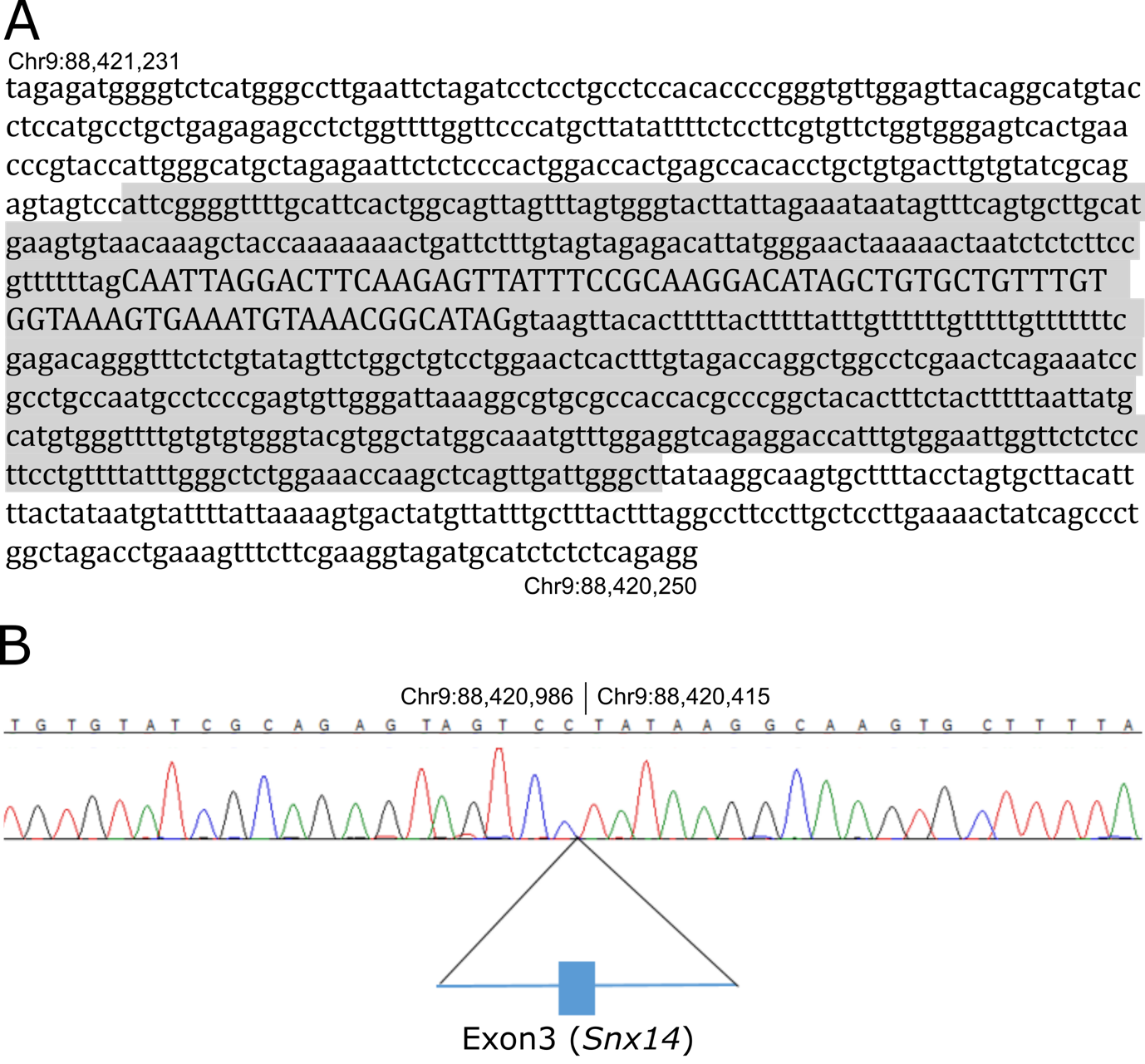


**Figure S1: Sequence of region targeted to generate knockout C57BL/6J** ***Snx14* allele in mice.** Illustration of mouse nucleotide sequence from position chr9:88,421,231 to 9:88,420,250 (GRCm38.p6). (A) Exon 3 of the mouse *Snx14* gene (ENSMUSE00001294503, chr9:88,420,825 – 88,420,749) is capitalised. The sequence coloured in grey marks the deleted 571bp nucleotide region (chr9:88,420,985 – 88,420,414). (B) Sanger sequencing showing deletion of this region with a continuous sequence jumping from position chr9:88,420,986 to chr9:88,420,415.


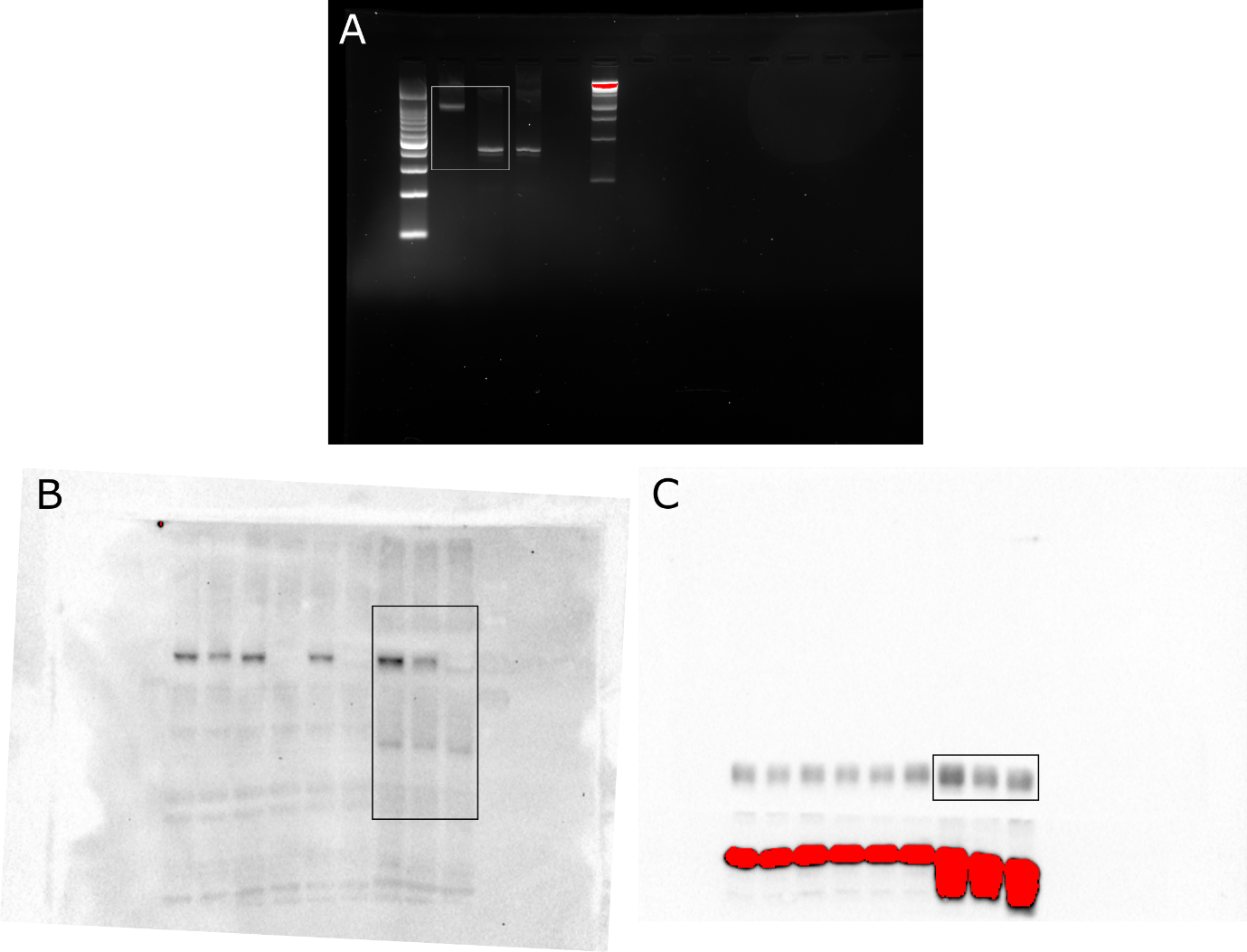


**Figure S2. Original gels and blots to accompany Figure 1.** A) Agarose gel of PCR products depicting alleles for *Snx14* WT and KO, showing the region cropped for Figure 1C. B) Western blot with anti-SNX14 showing the region cropped for Figure 1D (adjacent lanes were human cell lines included on the blot as controls for the antibody). C) Western blot with anti-Tubulin (top band) cropped for Figure 1D (GAPDH is the lower band).


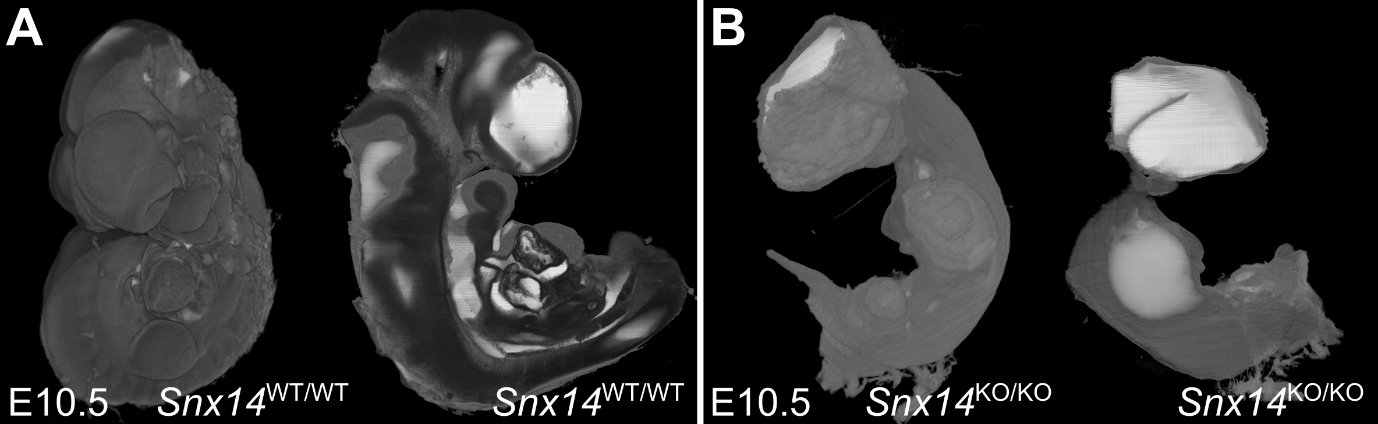


**Figure S3:** (A) *Snx14*^WT/WT^ and (B) *Snx14*^KO/KO^ E10.5 embryos visualised using high resolution episcopic microscopy (HREM).


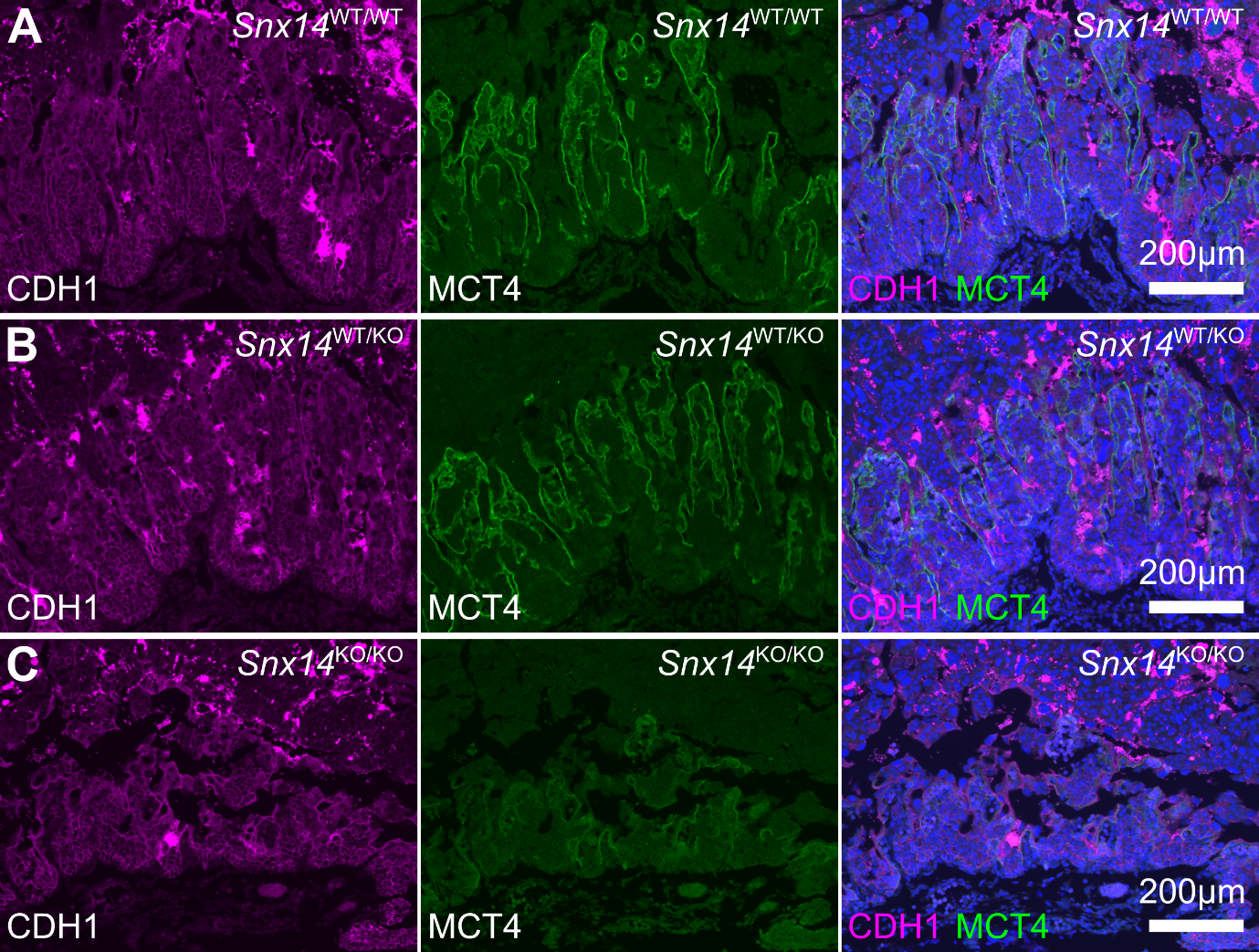


**Figure S4: The placentas of *Snx14*^WT/KO^ mice are comparable to the placentas of *Snx14*^WT/WT^ mice.** CDH1 (Magenta) and MCT (Green) immunoreactivity in the placentas of (A) *Snx14*^WT/WT^, (B) *Snx14*^WT/KO^, and (C) *Snx14*^KO/KO^ E10.5 mice. In the merged panels, CDH1 and MCT4 are combined with DAPI (Blue).


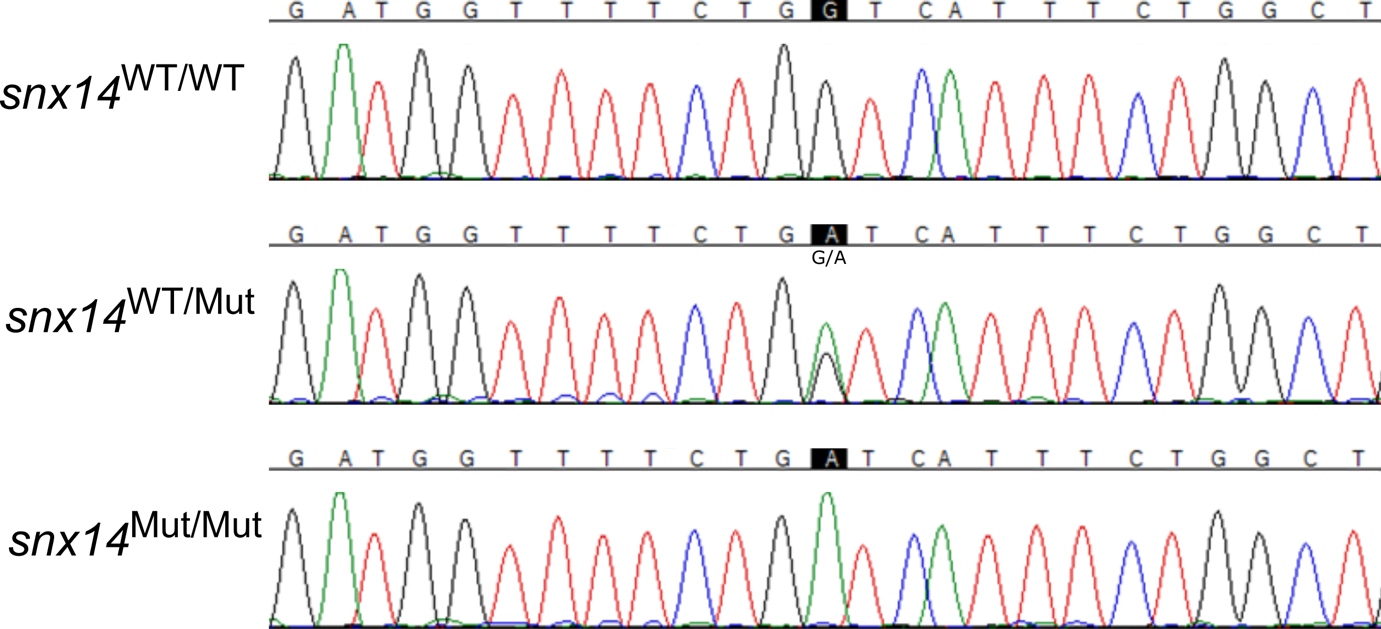


**Figure S5: Sequence of point mutation in *snx14* mutant zebrafish.** Sanger sequencing of *snx14* mutant zebrafish model (sa18413), created by ENU mutagenesis with a truncating G>A point mutation in exon 3 (F55*) of the *snx14* gene was obtained from the European Zebrafish Resource Center (EZRC).

**
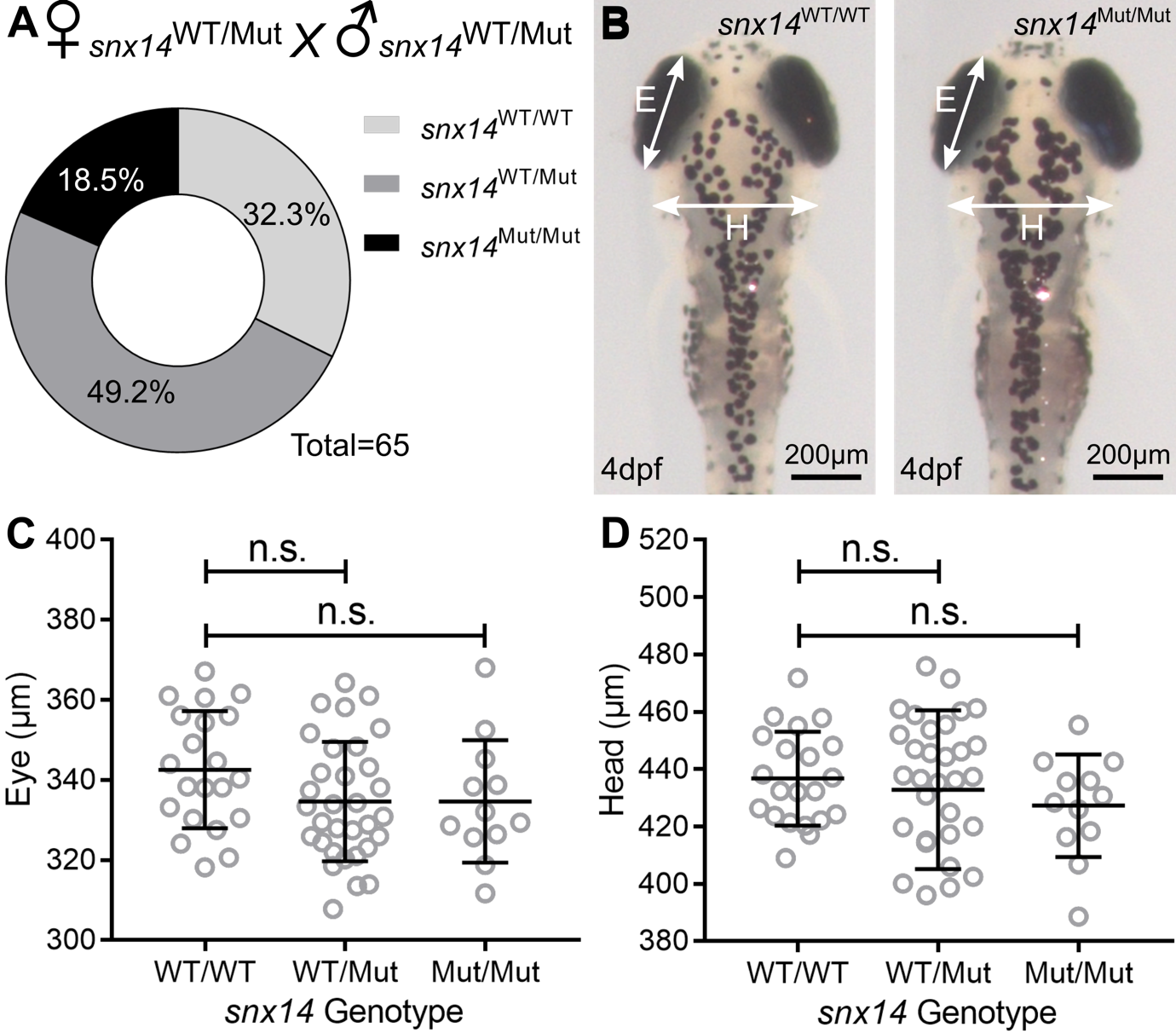
**

**Figure S6: Morphology of 4dpf zebrafish eye and tectum in offspring derived from breeding a *snx14*^WT/Mut^ female with *a snx14*^WT/Mut^ male.** (A) Ratio of *snx14* genotype in litters derived from *snx14*^WT/Mut^ female and *snx14*^WT/Mut^ male pairs. (B) Illustration and demonstration of zebrafish eye (E) width and head (H) width measurements (white arrows). (C) homozygous *snx14* mutations do not impact eye width. (D) homozygous *snx14* mutations do not impact head width. *N*≥12, circles = individual fish values, bars = mean, error bars = SD, n.s. (*p* ≥ 0.05), one-way ANOVA.


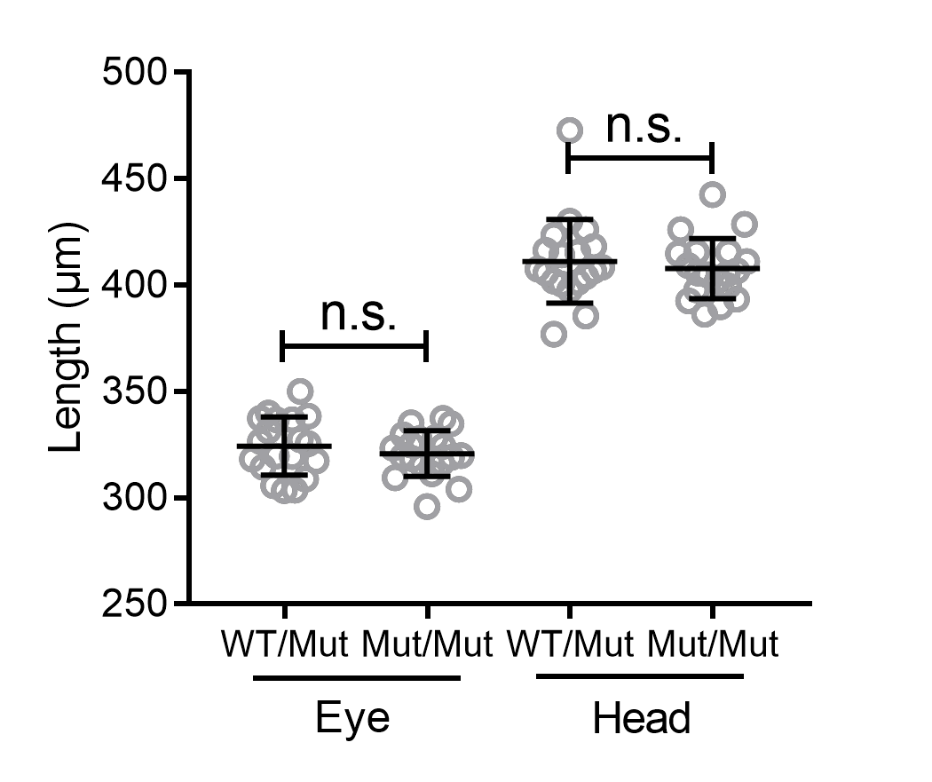


**Figure S7: MZ*snx14^Mut/Mut^* mutations do not impact eye or head width.** Analysis of fish derived from *snx14*^Mut/Mut^ female and *snx14*^WT/Mut^ male pairs The eye and head width was measured as described in figure 4. *N* = 19, circles = individual fish values, bars = mean, error bars = SD, n.s. (*p* ≥ 0.05), one-way ANOVA.

***
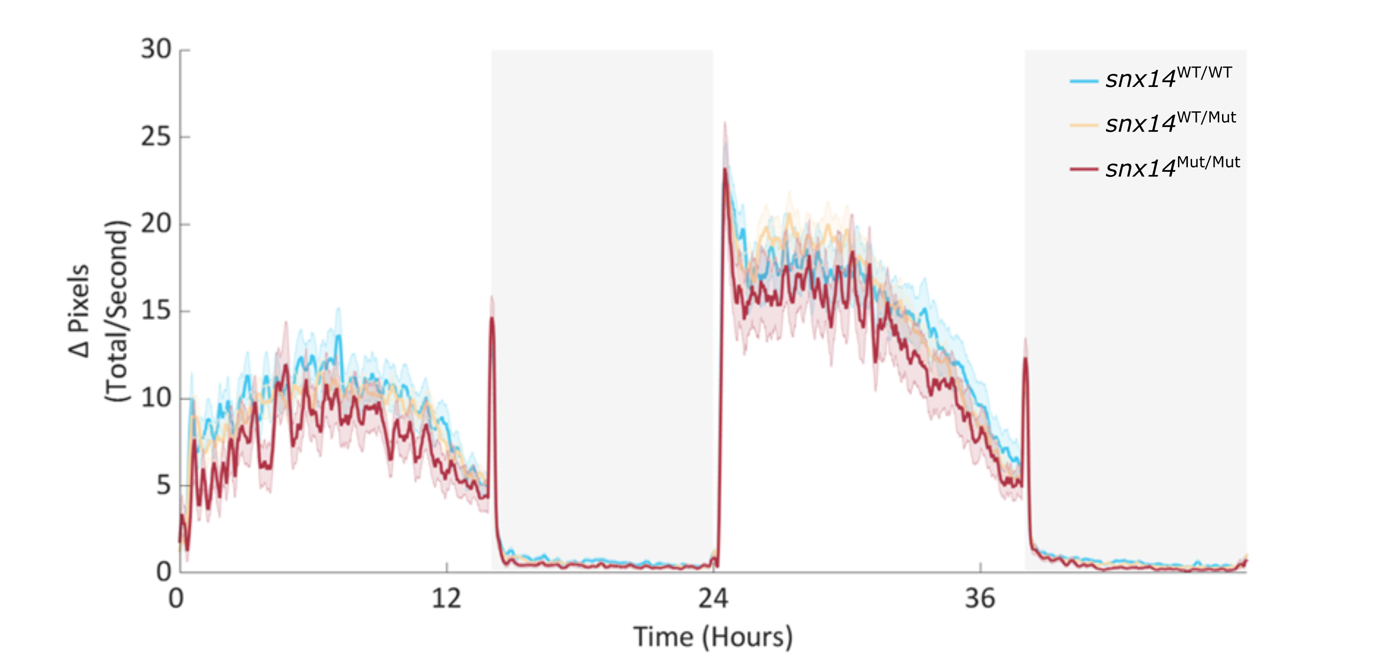
***

**Figure S8: Locomotor activity is not affected in *snx14* mutant zebrafish larvae at 4dpf.**

The mean activity of *snx14* wild type and mutant larvae across two 14hr/10hr light/dark cycles (day: white background; night: grey backgound). Delta pixel data for each larva was summed into seconds and then smoothed with a 15-minute running average. Shown per genotype is a mean trace (bold line) and standard error of the mean (shaded surround). *snx14^WT/WT^* (n = 56), *snx14^WT/Mut^* (n = 88) and *snx14^Mut/Mut^* (n = 30).


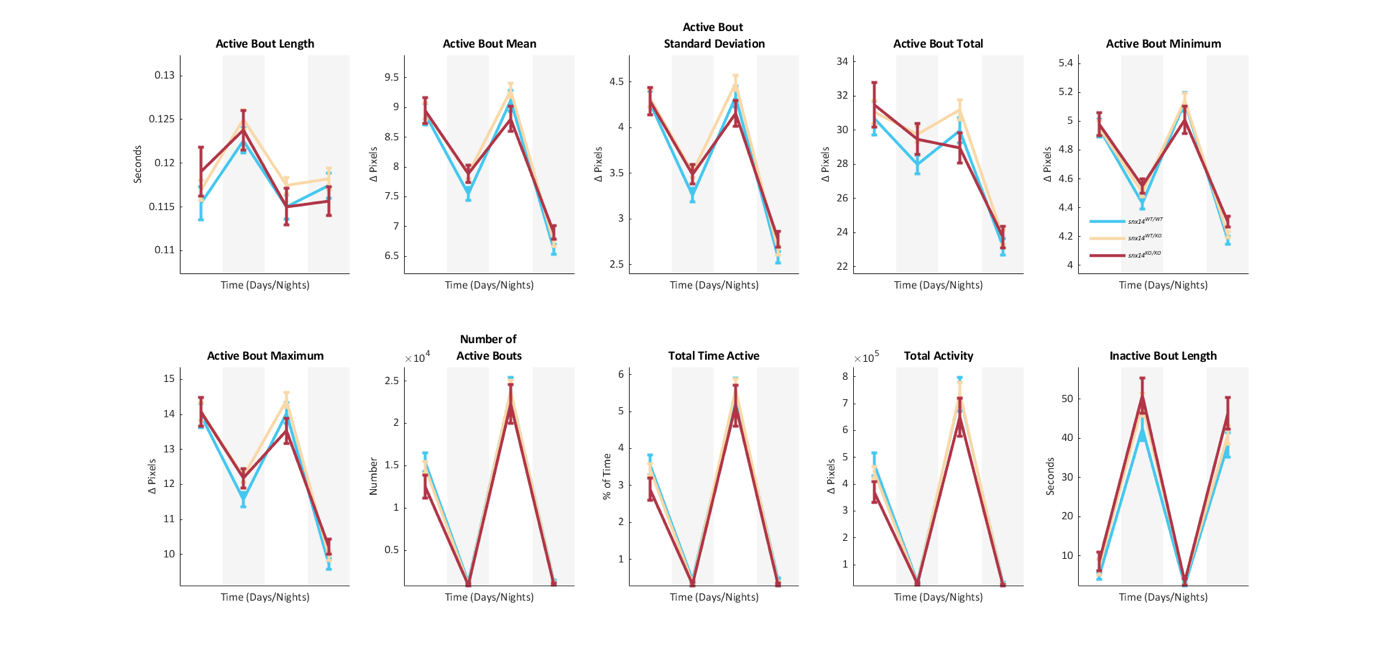


**Figure S9: Bout features of *snx14* mutants are indistinguishable from wild type at 4dpf.**

Shown are the means and standard error of the mean across two days (white background) and nights (grey background) for features of locomotor bout structure. Features were compared using a four-way ANOVA including the following factors: genotype, day/night, development and experimental repeat. No features or interaction terms were significantly different between genotypes. *snx14^WT/WT^* (n = 56, blue), *snx14^WT/Mut^* (n = 88, yellow) and *snx14^Mut/Mut^* (n = 30, red).

**Table S1: sgRNA target sequences used for the generation of *Snx14* mouse mutants.** sgRNAs were pooled together for targeting the *Snx14* gene in C57BL/6J and C57BL/6N mice.

| sgRNA | C57BL/6J (571bp deletion) | PAM | C57BL/6N (585bp deletion) | PAM |
| --- | --- | --- | --- | --- |
| #1 | 5’-ATCGCAGAGTAGTCCATTCG-3’ | GGG | 5’-ATCGCAGAGTAGTCCATTCG-3’ | GGG |
| #2 | 5’-GTATCGCAGAGTAGTCCATT-3’ | CGG | 5’-GTATCGCAGAGTAGTCCATT-3’ | CGG |
| #3 | 5’-CTCAGTTGATTGGGCTTATA-3’ | AGG | 5’-GTAGAAAGTGTAGCCGGGCG-3’ | TGG |
| #4 | 5’-TAAGCCCAATCAACTGAGCT-3’ | TGG | 5’-CTTTAATCCCAACACTCGGG-3’ | AGG |

**Table S2: Frequency of *Snx14* allele combinations at different developmental stages in** C57BL/6J and C57BL/6NTac mice**.** Frequency of *Snx14*^WT^ and *Snx14*^KO^ allele combinations generated from breeding mice that are both heterozygous for the *Snx14*^KO^ allele.

|  | C57BL/6J *Snx14*^KO^  (DEL571) | | | C57BL/6N *Snx14*^KO^ (DEL585) |
| --- | --- | --- | --- | --- |
|  | **E10.5** | **E12.5** | **P0** | **E12.5** |
| *Snx14*^WT/WT^ | 18 (27%) | 13 (35%) | 10 (38%) | 3 (19%) |
| *Snx14*^WT/KO^ | 38 (58%) | 21 (57%) | 16 (62%) | 13 (81%) |
| *Snx14*^KO/KO^ | 10 (15%) | 3 (8%) | 0 (0%) | 0 (0%) |
| Total | 66 | 37 | 26 | 16 |
| Reabsorption | 15 | 9 | NA | 7 |

**Table S3: Zebrafish lipidomic data**

| **Lipid** | **Species*** | ***snx14*^WT/WT^** | ***snx14*^WT/Mut^** | ***snx14*^Mut/Mut^** |
| --- | --- | --- | --- | --- |
| Neutral Lipid | FA(16:0) | 37.17±1.65 | 33.05±4.02 | 49.59±2.04 |
| Neutral Lipid | FA(18:0) | 22.22±1.12 | 27.27±2.59 | 25.50±0.41 |
| Neutral Lipid | FA(18:1(n9)) | 10.50±0.40 | 9.07±1.87 | 22.54±0.48 |
| Neutral Lipid | FA(18:2(n6)) | 6.80±0.13 | 6.31±0.60 | 11.74±0.25 |
| Neutral Lipid | FA(20:4(n6)) | 0.65±0.13 | 0.56±0.12 | 1.46±0.09 |
| Neutral Lipid | FA(22:6(n3)) | 4.49±0.34 | 4.91±0.30 | 14.49±0.20 |
| Phospholipid | FA(16:0) | 124.69±2.27 | 170.74±4.77 | 169.92±2.10 |
| Phospholipid | FA(18:0) | 76.36±0.75 | 90.06±2.68 | 92.57±1.35 |
| Phospholipid | FA(18:1(n9)) | 54.96±1.32 | 134.00±4.54 | 126.85±2.32 |
| Phospholipid | FA(18:2(n6)) | 20.10±0.82 | 49.94±1.82 | 46.27±0.90 |
| Phospholipid | FA(20:4(n6)) | 3.76±0.068 | 6.19±0.40 | 6.15±0.20 |
| Phospholipid | FA(22:6(n3)) | 32.59±2.36 | 131.02±9.15 | 71.78±2.42 |
| Total Lipid | FA(16:0) | 161.86±2.38 | 203.79±8.78 | 219.51±4.14 |
| Total Lipid | FA(18:0) | 98.58±1.87 | 117.32±5.27 | 118.07±1.75 |
| Total Lipid | FA(18:1(n9)) | 65.46±1.69 | 144.07±5.99 | 149.39±2.80 |
| Total Lipid | FA(18:2(n6)) | 26.90±0.95 | 56.249±2.41 | 58.003±1.15 |
| Total Lipid | FA(20:4(n6)) | 4.41±0.11 | 6.757±0.42 | 7.603±0.12 |
| Total Lipid | FA(22:6(n3)) | 37.08±2.32 | 135.93±9.10 | 86.267±2.52 |

*Fatty Acid (number of C-atoms:number of double bonds(double bonds position))
